# C-Reactive Protein Stimulates Nicotinic Acetylcholine Receptors to Control ATP-Mediated Monocytic Inflammasome Activation

**DOI:** 10.3389/fimmu.2018.01604

**Published:** 2018-07-30

**Authors:** Katrin Richter, Sabrina Sagawe, Andreas Hecker, Mira Küllmar, Ingolf Askevold, Jelena Damm, Sarah Heldmann, Michael Pöhlmann, Sophie Ruhrmann, Michael Sander, Klaus-Dieter Schlüter, Sigrid Wilker, Inke R. König, Wolfgang Kummer, Winfried Padberg, Arik J. Hone, J. Michael McIntosh, Anna Teresa Zakrzewicz, Christian Koch, Veronika Grau

**Affiliations:** ^1^Laboratory of Experimental Surgery, Department of General and Thoracic Surgery, Justus-Liebig-University Giessen, German Centre for Lung Research (DZL), Giessen, Germany; ^2^Department of Anesthesiology and Intensive Care Medicine, Justus-Liebig-University Giessen, Giessen, Germany; ^3^Physiological Institute, Justus-Liebig-University Giessen, Giessen, Germany; ^4^Institute of Medical Biometry and Statistics, University of Luebeck, Luebeck, Germany; ^5^Airway Research Center North (ARCN), German Center for Lung Research (DZL), Giessen, Germany; ^6^Institute of Anatomy and Cell Biology, Justus-Liebig-University Giessen, German Centre for Lung Research, Giessen, Germany; ^7^Department of Biology, University of Utah, Salt Lake City, UT, United States; ^8^George E. Wahlen Veterans Affairs Medical Center, Salt Lake City, UT, United States; ^9^Department of Psychiatry, University of Utah, Salt Lake City, UT, United States

**Keywords:** C-reactive protein, interleukin-1β, NLRP3 inflammasome, monocytes, nicotinic acetylcholine receptors, sterile inflammation

## Abstract

Blood levels of the acute phase reactant C-reactive protein (CRP) are frequently measured as a clinical marker for inflammation, but the biological functions of CRP are still controversial. CRP is a phosphocholine (PC)-binding pentraxin, mainly produced in the liver in response to elevated levels of interleukin-1β (IL-1β) and of the IL-1β-dependent cytokine IL-6. While both cytokines play important roles in host defense, excessive systemic IL-1β levels can cause life-threatening diseases such as trauma-associated systemic inflammation. We hypothesized that CRP acts as a negative feedback regulator of monocytic IL-1β maturation and secretion. Here, we demonstrate that CRP, in association with PC, efficiently reduces ATP-induced inflammasome activation and IL-1β release from human peripheral blood mononuclear leukocytes and monocytic U937 cells. Effective concentrations are in the range of marginally pathologic CRP levels (IC_50_ = 4.9 µg/ml). CRP elicits metabotropic functions at nicotinic acetylcholine (ACh) receptors (nAChRs) containing subunits α7, α9, and α10 and suppresses the function of ATP-sensitive P2X7 receptors in monocytic cells. Of note, CRP does not induce ion currents at conventional nAChRs, suggesting that CRP is a potent nicotinic agonist controlling innate immunity without entailing the risk of adverse effects in the nervous system. In a prospective study on multiple trauma patients, IL-1β plasma concentrations negatively correlated with preceding CRP levels, whereas inflammasome-independent cytokines IL-6, IL-18, and TNF-α positively correlated. In conclusion, PC-laden CRP is an unconventional nicotinic agonist that potently inhibits ATP-induced inflammasome activation and might protect against trauma-associated sterile inflammation.

## Introduction

Interleukin-1β (IL-1β) is a pro-inflammatory cytokine of innate immunity that plays a seminal role in host defense (1). Secretion of monocytic IL-1β into the circulation in response to severe multiple trauma, however, may be more harmful than beneficial, as IL-1β is swept away from the site of inflammation, and high blood plasma levels can cause systemic inflammatory response syndrome (SIRS) and multi organ dysfunction syndrome (MODS) (2–5). Despite decades of intensive research, treatment of SIRS and MODS is mainly supportive, and mortality remains unacceptably high (5, 6).

IL-1β secretion normally requires two consecutive danger signals (5, 7–9). Ligands of toll-like receptors are typical first signals inducing pro-IL-1β synthesis, and extracellular ATP is a typical second signal that activates the ATP-sensitive P2X7 receptor (P2X7R), induces NLRP3 (NACHT, LRR, and PYD domains-containing protein 3) inflammasome assembly, caspase-1 activation, pro-IL-1β cleavage, and secretion of mature IL-1β (7–11). The secretion of IL-18 and high mobility group box 1 protein (HMGB1) also depends on inflammasome activation (7–13). Apart from the ATP-induced pathway typical for trauma-associated sterile inflammation, several alternative mechanisms of IL-1β maturation are activated during infection (1).

C-reactive protein (CRP) is a pentraxin (14, 15), mainly produced in the liver in response to elevated systemic levels of IL-1β and IL-6 (14, 15). Blood concentrations of this acute phase protein are a frequently used sensitive clinical marker for inflammation. In addition, slightly raised CRP levels correlate with cardiovascular disease and some disorders of the central nervous system (16–18). The biological functions of CRP are, however, highly disputed. CRP seems to play a vital role in humans, as its gene was conserved during evolution and there are no reports on CRP-deficient individuals (14). It has been proposed to be involved in the clearance of pathogens or apoptotic cells (14, 15), to induce pro-inflammatory cytokines (19, 20), and to play a pathogenic role in cardiovascular diseases (21). At least some of the pro-inflammatory effects are mediated by activated CRP that exposes binding sites for complement and immunoglobulins and is mainly found within inflamed tissues (22, 23). In contrast to the more pro-inflammatory functions, CRP induces high levels of the anti-inflammatory IL-1 receptor antagonist (24), and transgenic animals overexpressing human CRP are protected from inflammatory diseases including sepsis (25), alveolitis (26), arthritis (27), and atherosclerosis (28).

In the presence of Ca^2+^, CRP associates with phosphocholine (PC) and a range of more complex molecules containing a PC group at a 1:1 M ratio per CRP monomer (14, 15, 29). Our group recently demonstrated that phosphatidylcholines and their metabolites including free PC are efficient inhibitors of ATP-induced release of IL-1β from human monocytes by a mechanism that involves non-canonical metabotropic functions of nicotinic acetylcholine (ACh) receptors (nAChRs) (30–33).

Here, we demonstrate that purified human endogenous CRP (eCRP) efficiently inhibits the ATP-induced release of IL-1β from monocytic cells. We provide evidence that CRP presents PC to nAChRs and thus potentiates the effect of free PC. PC-laden CRP is hence a novel agonist of unconventional nAChRs that controls inflammasome activation by inhibiting the function of P2X7R.

## Materials and Methods

### U937 Cells

U937 cells (DSMZ, Braunschweig, Germany) were maintained in RPMI 1640 (Gibco by Life Technologies, Darmstadt, Germany) supplemented with 10% fetal calf serum (FCS, Biochrome, Berlin, Germany) and 2 mM l-glutamine (Gibco by Life Technologies) under 5% CO_2_ atmosphere at 37°C. Cells (1 × 10^6^ cells/ml) were seeded in 24-well plates, primed for 5 h with 1 µg/ml lipopolysaccharide (LPS) from *Escherichia coli* (L2654, Sigma-Aldrich, Deisenhofen, Germany) (30). BzATP [2′(3′)-*O*-(4-benzoylbenzoyl)adenosine 5′-triphosphate triethylammonium salt; 100 µM, Sigma-Aldrich] or nigericin (50 µM, Sigma-Aldrich) were added for another 30 min in the presence or absence of different concentrations of eCRP from human pleural fluid (Millipore, AG732), recombinant CRP (rCRP) produced in *E. coli* (Millipore, 236608), serum amyloid P (SAP; Millipore, 565190), or PC chloride calcium salt tetrahydrate (Sigma-Aldrich). Nicotinic antagonists mecamylamine hydrochloride (Sigma-Aldrich), strychnine hydrochloride (Sigma-Aldrich), α-bungarotoxin (Tocris Bioscience, Bristol, UK), ArIB [V11L, V16D] (500 nM) (34, 35) and RgIA4 (200 nM) (31, 36) were also applied together with BzATP. Supernatants were stored at 20°C until cytokine and lactate dehydrogenase (LDH) measurement.

### Human Peripheral Blood Mononuclear Cells (PBMC)

Peripheral blood mononuclear cells were obtained from healthy (self-reported) male non-smoking adult volunteers. The local ethics committee at the University of Giessen approved all studies on primary human cells (approval No. 81/13). Blood was drawn into sterile syringes containing 17.5 IU heparin (Ratiopharm, Ulm, Germany) per ml blood and PBMC were separated on Leucosep gradients (Greiner Bio-One, Frickenhausen, Germany). LPS (5 ng/ml) was added to blood samples before gradient centrifugation (30). PBMC were cultured in 24-well plates at a density of 5 × 10^5^ cells/0.5 ml in RPMI 1640, 10% FCS, 2 mM l-glutamine for 3 h. Non-adherent cells were removed, and cell culture medium was replaced by medium devoid of FCS. Stimulation with BzATP in the presence or absence of eCRP was done as described for U937 cells.

### Cell Viability

Non-Radioactive Cytotoxicity Assay (Promega, Madison, WI, USA) was used to measure LDH concentrations in cell free supernatants as indicated by the supplier. LDH values are given as percentage of the total LDH content of lysed control cells. Cell viability was unimpaired in all experimental settings.

### Cytokine Measurement

Blood concentrations of IL-1β, IL-18, and tumor necrosis factor-α (TNF-α) were measured by the Human Quantikine^®^ Immunoassays (R&D Systems, Minneapolis, MN, USA). IL-6 was measured on the Siemens 150.

Immulite 2000 XPI system using the Siemens IL-6 reagent (Siemens, Erlangen, Germany). HMGB1 was measured by an ELISA obtained from IBL International (Hamburg, Germany). To detect low cytokine levels in cell culture supernatants, for IL-1β the Human IL-1 beta/IL-1F2 DuoSet ELISA (R&D Systems) was used, whereas IL6 and TNF-α were measured by the Human Quantikine^®^ Immunoassays (R&D Systems, Minneapolis, MN, USA).

### Dissociation and Formation of CRP/PC Complexes

Endogenous CRP was dissolved at a concentration of 5 µg/ml in PBS devoid of Ca^2+^ and Mg^2+^ (Gibco) containing 1.1 mM ethylenediaminetetraacetic acid (EDTA; Sigma-Aldrich), incubated at 37°C for 15 min followed by ultrafiltration using Amicon^®^ Ultra centrifugal filters. The high molecular weight fraction was diluted in PBS/EDTA, ultrafiltrated, and transferred to PBS, 5 mM Ca^2+^, without EDTA by two additional ultrafiltration steps. In control, the same procedure was performed in the absence of EDTA. CRP purified by ultrafiltration and rCRP were incubated at a 1:1 and 1:3 M ratio per monomer, respectively, with PC at 37°C for 30 min and tested in IL-1β release assays at a concentration of 5 µg/ml CRP and 1 µM PC.

### Gene Silencing

The expression of nAChR subunits α7 (*CHRNA7*), α9 (*CHRNA9*), and α10 (*CHRNA10*) in U937 cells was silenced by transfection of small interfering RNA (siRNA; 30 pmol per 1 × 10^6^ cells, ON-TARGETplus human *CHRNA7, CHRNA9*, or *CHRNA10* siRNA SMARTpool, Thermo Fisher Scientific, Schwerte, Germany) using the Amaxa^®^ Cell line Nucleofector^®^ Kit C (Lonza Cologne AG, Cologne, Germany) and the Nucleofector^®^ device II (Lonza Cologne AG). Negative control ON-TARGETplus non-targeting pool (Thermo Fisher Scientific) was included to control for non-specific effects of transfection. A reduction of the mRNA expression of subunits α9 and α10 to about 50% of control-transfected cells was recently shown by our group in the same experimental setting *via* real-time RT-PCR 6 h after transfection (30). The basal expression of α7 mRNA, however, was too low to be quantified. IL-1β release experiments were performed 2 days after transfection.

### Immunocytochemistry

Lipopolysaccharide-primed PBMC were cultured in CELLview™ slides (Greiner Bio-One) at a density of 2 × 10^5^ cells per well in 200 µl medium and stimulated with BzATP (100 µM) in the presence or absence of eCRP (5 µg/ml). Cells were fixed and permeabilized with ice-cold Cytofix/Cytoperm™ (BD Biosciences, Heidelberg, Germany) for 20 min, washed with Perm/Wash™ buffer (BD Biosciences), and air-dried before storage at 4°C. Slides were rehydrated with Perm/Wash™ buffer, endogenous peroxidase activity was inhibited by treatment with 1% H_2_O_2_ in Perm/Wash™ buffer, followed by 1% bovine serum albumin in Perm/Wash™ buffer for 30 min at ambient temperature. Polyclonal rabbit antibodies to human ASC (1:50, SC-22514-R, Santa Cruz Biotechnology, Dallas, TX, USA) or monoclonal mouse antibodies to human CD14 (1:100, HCD14, BioLegend *via* Biozol, Eching, Germany) were diluted in Perm/Wash™ buffer containing 1% bovine serum albumin and 5% human heat-inactivated serum. Bound antibodies were detected with horseradish peroxidase-conjugated goat anti-rabbit Ig (1:50) and rabbit anti-mouse Ig (1:70) antibodies (both from Dako Cytomation, Glostrup, Denmark) and 0.5 mg/ml 3,3′-diaminobenzidine (Sigma-Aldrich), 1% H_2_O_2_, 0.3 M Tris-buffered saline, pH 7.6, for 10 min at room temperature. Slides were slightly counterstained with hemalumn and cover-slipped in Glycergel mounting medium (Dako Cytomation). Slides were evaluated blinded for the experimental groups at a 200-fold magnification using an Olympus BX51 microscope and the analySIS software (Olympus, Hamburg, Germany). At least 150 cells in 12 fields of vision were counted per experiment. Total numbers of cells and specks were converted into mean numbers of specks per 100 cells. In negative controls that resulted in no staining, primary antibodies were omitted. The specificity of the antibodies to ASC was verified by Western blotting of protein extracts from U937 cells that revealed a single band with the expected molecular mass (not shown).

### Gel Electrophoresis and Western Blotting

SDS polyacrylamide gel electrophoresis was performed under reducing conditions according to Laemmli (37). PBMC were lysed, the protein concentration of the lysate was determined (Micro BCA protein assay kit, Pierce Biotechnology, Rockford, IL, USA) and adjusted to 15 μg/10 μl. Cell culture supernatants were harvested at the end of the experiments, concentrated by a factor of 10 using Amicon^®^ Ultra centrifugal filters (Ultracel™ 10K, Merck Millipore, Darmstadt, Germany) and mixed with 2× sample buffer. Samples (10 µl each) were loaded onto 15% SDS-polyacrylamide gels, transferred to polyvinylidene difluoride membranes (Millipore, Billerica, MA, USA) and stained with Brilliant Blue G (Sigma-Aldrich). Prestained molecular weight standards (Precision Plus Protein Standards, dual color, Bio Rad, Hercules, CA, USA) were separated in each gel. Mouse monoclonal antibodies to IL-1β that detect both pro-IL-1β and mature IL-1β (1:10,000, 3ZD, kindly supplied by the National Cancer Institute, Frederick, MD, USA), polyclonal rabbit anti-caspase-1 antibodies (1:1,000, #2225, Cell Signaling Technology, Danvers, MA, USA) and mouse monoclonal antibodies to β-actin (1:500,000, A2228, Sigma-Aldrich) were applied and detected with horseradish peroxidase-conjugated rabbit anti-mouse Ig (1:5,000) and goat anti-rabbit Ig (1:5,000) antibodies (both from Dako Cytomation). SuperSignal West Dura Extended Duration Substrate (Thermo Scientific, Rockford, IL, USA) was used to detect IL-1β and Lumi-Light substrate (Roche, Mannheim, Germany) to detect β-actin. Documentation and densitometry of the blots were performed using a digital gel documentation system (Biozym, Hessisch Oldendorf, Germany).

### Whole-Cell Patch-Clamp Recordings

U937 cells were incubated in poly-l-lysine-coated culture dishes (Nunc, Roskilde, Denmark) in bath solution [5.4 mM KCl, 120 mM NaCl, 2 mM CaCl_2_, 1 mM MgCl_2_, 10 mM HEPES (4-(2-hydroxyethyl)-piperazine-1-ethanesulfonic acid), 25 mM glucose, pH 7.4] for 5 h with LPS (1 µg/ml) at 37°C. Thereafter, whole-cell recordings were performed at ambient temperature on an inverted microscope (Axiovert, Zeiss, Göttingen, Germany). Patch pipettes were pulled from borosilicate glass capillaries (outer diameter 1.6 mm, Hilgenberg, Malsfeld, Germany) to a resistance of 2–4 MΩ using an automated puller (Zeitz, Augsburg, Germany). Pipettes were filled with pipette solution (120 mM KCl, 1 mM CaCl_2_, 2 mM MgCl_2_, 10 mM HEPES, 11 mM ethylene glycol tetraacetic acid, 20 mM glucose, pH 7.3). The membrane potential of LPS-primed U937 cells was voltage-clamped to −60 mV and transmembrane currents in response to BzATP (100 µM) were amplified with an EPC 9 amplifier (HEKA, Lambrecht, Germany) and acquired *via* an ITC-16 interface with the Pulse software (HEKA). A pressure-driven microperfusion system was used to apply BzATP, eCRP (5 µg/ml) and RgIA4 (200 nM).

### Measurements of Intracellular Ca^2+^

To measure intracellular [Ca^2+^]_i_, U937 cells were incubated in poly-l-lysine-coated glass bottom culture dishes (CELLview™, Greiner Bio-One) for 5 h with LPS (1 µg/ml) at 37°C in the same bath solution as described for the whole-cell patch-clamp recordings. Thereafter, cells were loaded with 3.3 µM Fura-2/AM (Thermo Fisher Scientific) for 25 min at 37°C. Fura-2/AM was excited at 340 and 380 nm wavelengths and the fluorescence emission 510 nm was measured. Four independent batches of U937 cells were used in this experiment and a total number of 243 cells were tracked individually, and the fluorescence intensity ratio of 340:380 nm was recorded. Experiments were run at room temperature. After a calibration time of 100 s, cells were exposed to eCRP (5 µg/ml) for 300 s. At the end of the experiments, a positive control for the Ca^2+^ imaging setup was included. Forskolin (40 µM; Biozol), an activator of adenylyl cyclase that elevates cyclic adenosine monophosphate (cAMP) levels, was applied to induce a cAMP-triggered rise in [Ca^2+^]_i_ (38).

### Two-Electrode Voltage-Clamp (TEVC) Measurements on *Xenopus laevis* Oocytes Expressing Human nAChR

Defolliculated *Xenopus laevis* oocytes were obtained from Ecocyte Bioscience (Castrop-Rauxel, Germany). Oocytes from at least two different *Xenopus laevis* individuals were used in all experimental groups. Oocytes were stored in Ringer’s solution (ORi) containing (in mM) 90 NaCl, 1 KCl, 2 CaCl_2_, 5 HEPES, 2.5 pyruvate, 20 mg/ml penicillin, and 25 mg/ml streptomycin (pH 7.4) at 16°C. All chemicals used for ORi preparation were purchased from Fluka (Deisenhofen, Germany), except for HEPES, penicillin, and streptomycin (Sigma-Aldrich). Plasmid DNAs encoding the human *CHRNA9* and *CHRNA10* as well as the 43 kDa receptor-associated protein of the synapse (*RAPSN*) were obtained from Eurofins Genomics (Ebersberg, Germany) and capped cRNA was synthesized as described before (31). Human *CHRNA7* encoding cRNA was kindly provided by G. Schmalzing (Department of Molecular Pharmacology, RWTH Aachen University, Aachen, Germany) and synthesized as described before (33). cRNA was dissolved in nuclease-free water and injected into oocytes in a volume of 50.6 nl using a microinjector (Nanoject, Drummond Scientific, Broomall, PA, USA). Oocytes were injected with cRNA encoding *CHRNA7, CHRNA9*, and *CHRNA10* nAChR subunits (16, 19, and 19 ng/oocyte, respectively) along with 5 ng cRNA encoding *RAPSN*, and oocytes were incubated at 16°C for 3–5 days. In control experiments, 50.6 nl of nuclease-free water was injected.

In TEVC measurements, oocytes were perfused (gravity driven) with ORi without pyruvate and antibiotics (pH 7.4). Intracellular borosilicate microelectrodes were filled with 1 M KCl solution and the membrane voltage was clamped to –60 mV using a TEVC amplifier (Warner Instruments, Hamden, CT, USA). Low-pass filtered transmembrane currents (1,000 Hz, Frequency Devices 902, Haverhill, MA, USA) were recorded using a strip chart recorder (Kipp & Zonen, Delft, The Netherlands). For experiments examining the inhibition and recovery from inhibition of choline-gated currents in presence and absence of eCRP (5 µg/ml), oocytes were injected with a 1:1 ratio of cRNA encoding human *CHRNA9* and *CHRNA10* in a 1:1 ratio, incubated at 17°C for 2–3 days and measured as described before (31).

### Clinical Study

A single center prospective observational study (trial registration: DRKS00010991) was approved by the ethics committee of the medical faculty Giessen, Germany (No. 164/14) and performed in accordance with the Helsinki Declaration. Written informed consent was given by each patient or patient’s legal representative. Patients were recruited at the surgical intensive care unit (ICU) of the University Hospital of Giessen, Germany from January 2015 until February 2016. Only patients with severe trauma as defined by an injury severity score (ISS) above 16 (39) were included. Patients younger than 18 years or with a history of HIV or hepatitis B/C infection were excluded. Detailed patient characteristics are listed in Table 1.

**Table 1:** Patient characteristics.

Number of patients	*n* = 38
Gender male	28 (73.7%)
Age (years)	47.7 ± 20.9
Body mass index (kg/m^2^)	25.3 ± 3.3
**Clinical history**	
COPD	0
Myocardial infarction	1 (2.6%)
Congestive heart failure	0
Renal failure	0
Immunosuppression	0
ISS	25 ± 6
NISS	27 ± 6
**Type of injury**	
Head	24 (63.2%)
Thorax	34 (89.5%)
Abdomen	24 (63.2%)
Extremities	28 (73.7%)
External injuries	35 (92.1%)
**Cause of injury**	
Car accident	16 (42.1%)
Motorcycle accident	10 (26.3%)
Bicycle accident	3 (7.9%)
Fall	7 (18.4%)
Other reasons	2 (5.3%)
RISC	2.6 ± 1.3
APACHE II	16.8 (7.5%)
SOFA	7.2 (4.3%)
24 h mortality	1 (2.6%)
30 days mortality	2 (5.3%)
Length of ICU stay (days)	11.2 ± 11.8
Length of hospital stay (days)	23.5 ± 15.2

*APACHE II, acute physiology and chronic health evaluation score; COPD, chronic obstructive pulmonary disease; ICU, intensive care unit; ISS, injury severity score; NISS, new injury severity score; RISC, revised injury severity classification score; SOFA, sequential organ failure assessment score*.

*Data are shown as mean ± SD, as absolute numbers or as percentage*.

The first venous blood sample (day 0) was drawn within 15 h after admission to the hospital followed by daily blood collection in the morning. The levels of IL-1β, IL-18, TNF-α, IL-6, and HMGB1 were determined in the plasma of heparinized blood. CRP levels were analyzed turbidometrically by Siemens ADVIA XPT system (Siemens) using the wrCRP reagent (Siemens).

### Quantification and Statistical Analysis

SPPS^®^ (Version 23, IBM^®^, Armonk, NC, USA) and GraphPad Prism^®^ (Version 6, GaphPad Software, La Jolla, CA, USA) were used for statistical and linear regression analyses. The IC_50_ value of eCRP in human U937 cells was determined in GraphPad Prism^®^ (Version 6, GaphPad Software) by fitting log-transformed concentration values and the original effect data. Multiple groups were first analyzed by non-parametric Kruskal–Wallis test. In case of *p* ≤ 0.05, non-parametric Mann–Whitney rank sum test was performed to compare between individual groups and again, a *p* ≤ 0.05 was considered as statistically significant. Paired data were analyzed by Wilcoxon sign rank test.

## Results

### CRP Inhibits ATP-Mediated IL-1β Release From Human Monocytic U937 Cells

**Figure 1:**
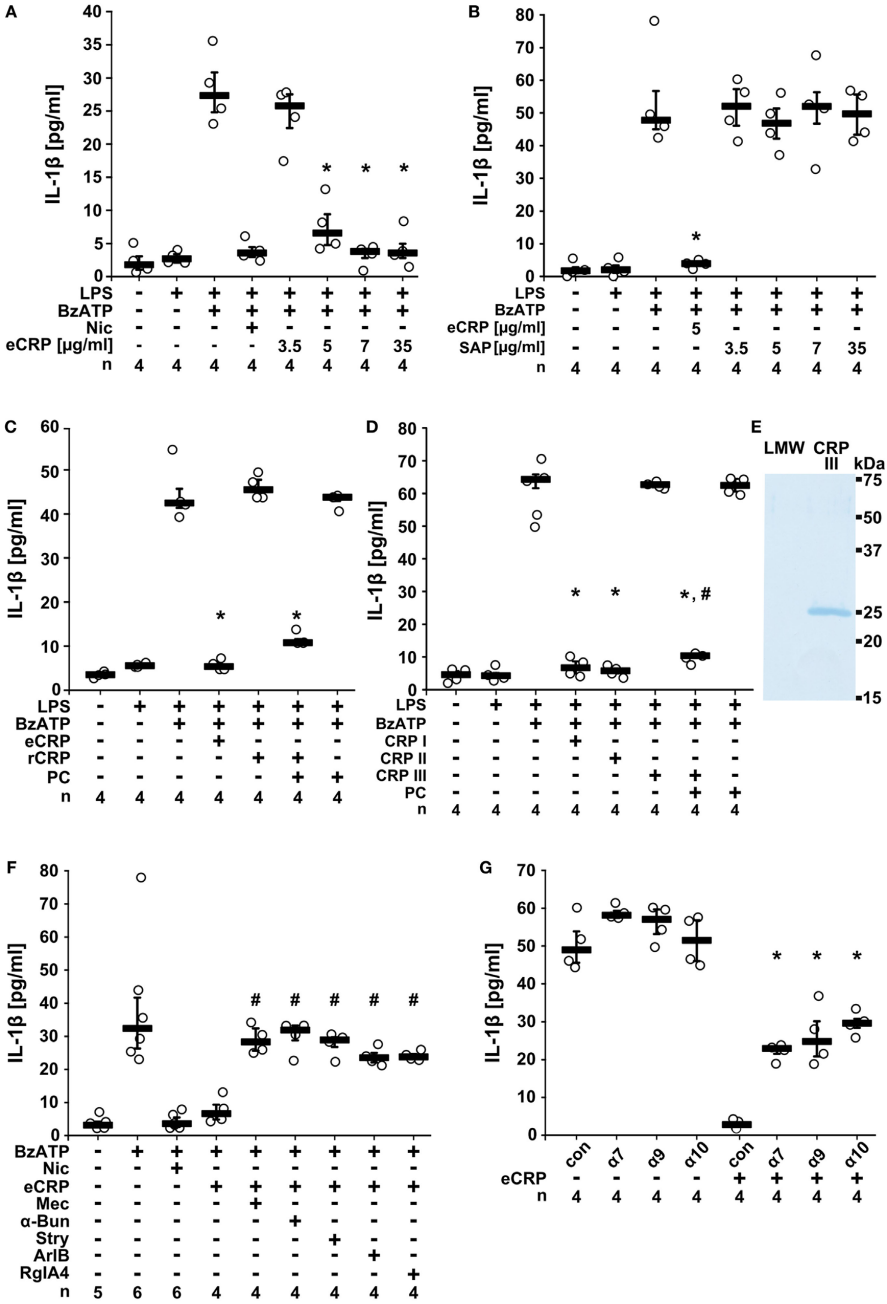
Purified human endogenous C-reactive protein (eCRP) inhibits BzATP-induced release of interleukin-1β (IL-1β) from U937 cells. Lipopolysaccharide (LPS)-primed (1 µg/ml, 5 h) U937 cells were stimulated with BzATP (2′(3′)-O-(4-benzoylbenzoyl)adenosine 5′-triphosphate triethylammonium salt; 100 µM) and IL-1β was measured 30 min later in cell culture supernatants. **(A)** eCRP dose-dependently inhibited the BzATP-induced IL-1β release, nicotine (Nic; 100 µM) served as a positive control. **(B,C)** Serum amyloid P (5 µg/ml), human recombinant CRP (rCRP) (5 µg/ml), or low concentrations of free phosphocholine (PC) (1 µM) did not impair IL-1β release, but a combination of rCRP and PC (1 µM) did. **(D)** The inhibitory effect of eCRP (CRP I; 5 µg/ml) was preserved after ultrafiltration (cutoff 10 kDa; CRP II), but abolished by ultrafiltration in the presence of ethylenediaminetetraacetic acid (1.1 mM; CRP III). PC (1 µM) reconstituted the activity of CRP III, whereas 1 µM PC alone was ineffective. **(E)** CRP was retained in CRP III and absent from the low molecular weight fraction (LMW). SDS-PAGE followed by staining with Brilliant Blue. **(F)** The effect of eCRP was reversed by nicotinic acetylcholine receptor (nAChR) antagonists mecamylamine (Mec; 100 µM), α-bungarotoxin (α-Bun; 1 µM), strychnine (Stry; 10 µM), ArIB (500 nM), and RgIA4 (200 nM). **(G)** In experiments using small interfering RNA (siRNA), silencing of the nAChR subunits α7, α9, and α10, but not control siRNA (con) attenuated the inhibition by eCRP. Data are presented as individual data points, bar represents median, whiskers encompass the 25th to 75th percentile. *p ≤ 0.05, different from LPS-primed cells stimulated with BzATP alone. ^#^*p* ≤ 0.05, different LPS-primed cells were stimulated with BzATP and eCRP. Kruskal–Wallis followed by Mann–Whitney rank sum test.

To test if CRP inhibits ATP-induced IL-1β release, we primed human monocytic U937 cells for 5 h with LPS (1 µg/ml). Thereafter, 100 µM BzATP, a P2X7R agonist (40), was applied for 30 min in presence and absence of eCRP and IL-1β concentrations were measured in cell culture supernatants. We found that in LPS-primed U937 cells the BzATP-induced release of IL-1β was dose-dependently and efficiently inhibited by eCRP (Figure 1A). The half maximal inhibitory concentration (IC_50_) was 4.9 µg/ml corresponding to an about 40 nM concentration of pentameric CRP. Of note, plasma levels between 3 and 10 µg/ml are clinically regarded as a minor pathological CRP elevation (41). In contrast to eCRP, the acute phase protein (5 µg/ml), another pentraxin with high structural similarity to CRP (15), did not inhibit ATP-induced IL-1β release (Figure 1B).

### CRP Potentiates the Inhibitory Effect of PC

C-reactive protein, but not SAP, Ca^2+^-dependently associates with PC in a 1:1 M ratio per subunit (15, 29). We previously showed that PC efficiently inhibits BzATP-induced IL-1β secretion from human monocytes (30, 31). Thus, we hypothesized that the inhibitory effect of eCRP on the release of IL-1β depends on its association with molecules containing a PC group. Accordingly, human rCRP (5 µg/ml) produced in *E. coli* that is expected to be devoid of PC, did not inhibit the BzATP-triggered release of IL-1β from LPS-primed U937 cells (Figure 1C), whereas a mixture of 5 µg/ml rCRP, 1 µM PC, and 5 mM Ca^2+^ was fully effective (Figure 1C). The same concentration of free PC was ineffective (Figure 1C), as expected (30). In a similar approach, eCRP was treated with EDTA (1.1 mM) to detach ligands from the PC-binding sites and was separated from small molecules by ultrafiltration (Figures 1D,E). This resulted in an inactive CRP preparation, the activity of which was restored by adding PC (1 µM) in the presence of Ca^2+^ (Figure 1D). Hence, the inhibition of ATP-induced IL-1β release by eCRP depends on its association with PC. Moreover, as the IC_50_ eCRP is around 40 nM and that of free PC around 10 µM (30, 31), CRP potentiates the inhibitory effect of PC by at least two orders of magnitude.

### eCRP Signals *via* nAChRs

Next, we tested if eCRP signals *via* nAChRs in human monocytic U937 cells. Indeed, the eCRP-dependent inhibition of ATP-induced IL-1β release was completely reversed by the non-selective nAChR antagonist mecamylamine (100 µM). Similar results were obtained by using α-bungarotoxin (1 µM) or strychnine (10 µM), both preferential antagonists of the α7 and α9 nAChRs (42–44). The α-conotoxins ArIB [V11L, V16D] (500 nM) (34, 35) and RgIA4 (200 nM) (31, 36) that are specific for nAChRs composed of subunits α7 and α9α10, respectively, also completely blocked the inhibitory effect of eCRP (Figure 1F). These results indicate that eCRP signals *via* nAChRs containing subunits α7, α9, and α10. To corroborate the involvement of these nAChR subunits, U937 cells were transfected with siRNA targeting *CHRNA7, CHRNA9*, and *CHRNA10*. Indeed, single-gene silencing of each nAChR subunit significantly blunted the inhibitory effect of eCRP (Figure 1G).

### eCRP Does Not Trigger Ion Channel Functions at Conventional nAChRs

**Figure 2:**
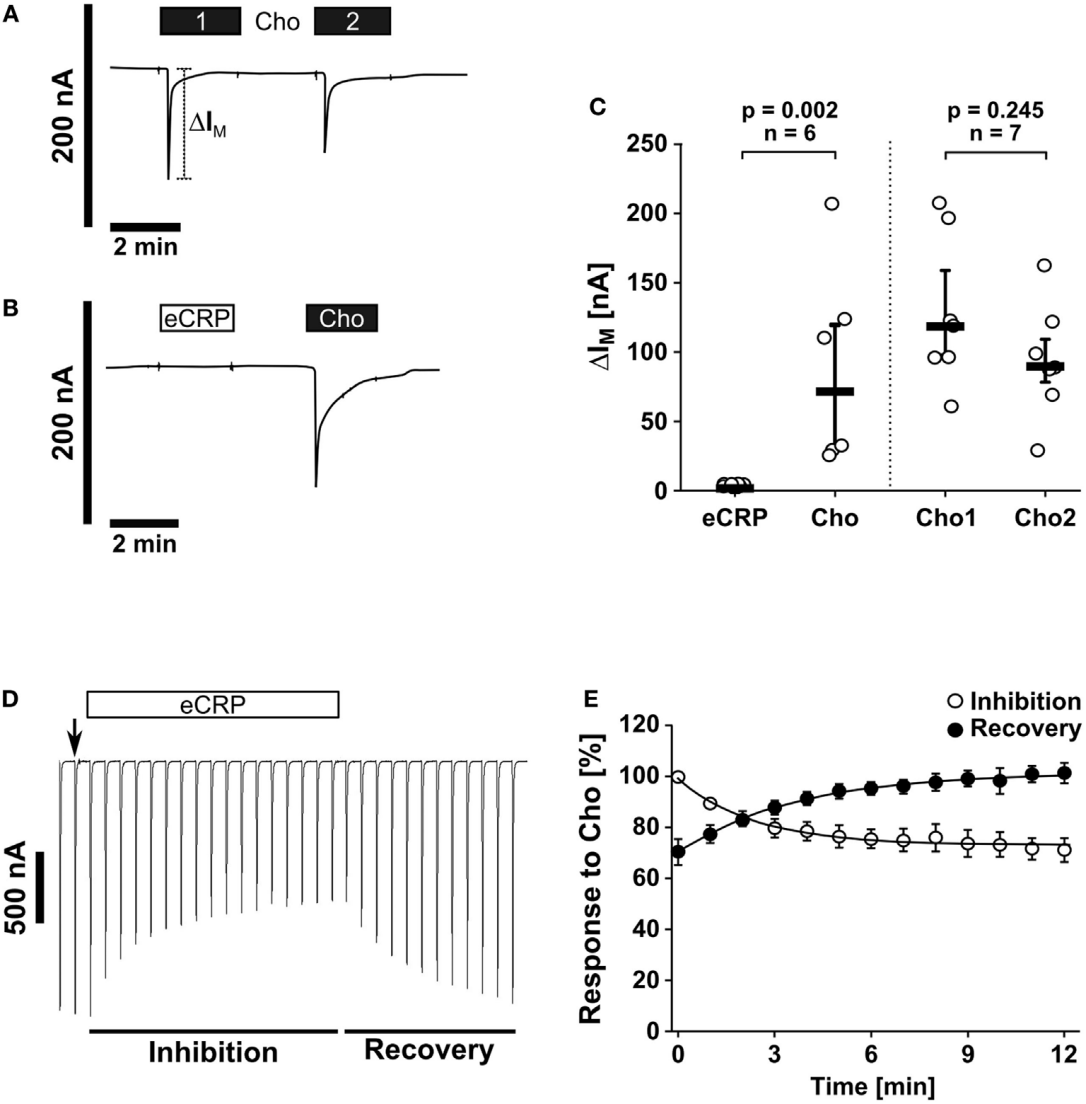
Purified human endogenous C-reactive protein (eCRP) does not induce ion channel functions at canonical nAChRs. Two-electrode voltage-clamp (TEVC) measurements were performed on *Xenopus laevis* oocytes that heterologously expressed human nAChR subunits α7, α9, and α10. **(A,C)** The application of the known nAChR agonist choline (Cho1, 2 min, 1 mM, black bars) induced a stimulation of the transmembrane ion current (I_M_) that could be repeated by a second Cho application (Cho2; *n* = 7). **(B,C)** By contrast, eCRP (5 µg/ml) did not provoke current responses, whereas a subsequent Cho application did (*n* = 6). **(C)** All changes of I_M_ (ΔI_M_) induced by cholinergic stimulation are shown as individual data points, bars represent median, whiskers encompass the 25th to 75th percentile. Wilcoxon signed-rank test. **(D)** Representative current traces of Cho-gated currents in oocytes expressing α9α10 nAChRs illustrating the inhibitory effect of eCRP (5 µg/ml). The current traces represent 30 s recordings each and are shown concatenated omitting the 30 s gap between each individual trace. The oocytes were continuously perfused with saline solution and stimulated with Cho (1 s pulses, 1 mM) once per min until steady-state baseline responses were observed (indicated by the arrow). Subsequently, the Cho-gated currents were monitored in the presence of eCRP for changes in amplitude for 12 min. Thereafter, eCRP was washed out and the Cho-gated currents were monitored for recovery. **(E)** Graphical representation and analysis of the experimental results shown in **(D)**. Mean and SEM from five oocytes.

The canonical ionotropic function of nAChR can be monitored by using *Xenopus laevis* oocytes as heterologous expression systems (45). To investigate if eCRP elicits ionfluxes human nAChR subunits α7, α9, and α10 were heterologously expressed in oocytes to perform TEVC measurements. We showed that eCRP (5 µg/ml) did not induce ion currents, whereas the classical nAChR agonist choline did (Figures 2A–C). Nevertheless, eCRP interacted with canonical nAChRs, as it reduced choline-triggered currents in oocytes expressing α9α10 nAChRs (Figures 2D,E). Thus, eCRP does not trigger canonical ion channel functions of nAChRs but acts as a silent agonist or partial antagonist that modulates the responses to classical nicotinic agonists.

### eCRP Inhibits BzATP-Induced Current Responses in Monocytic Cells

**Figure 3:**
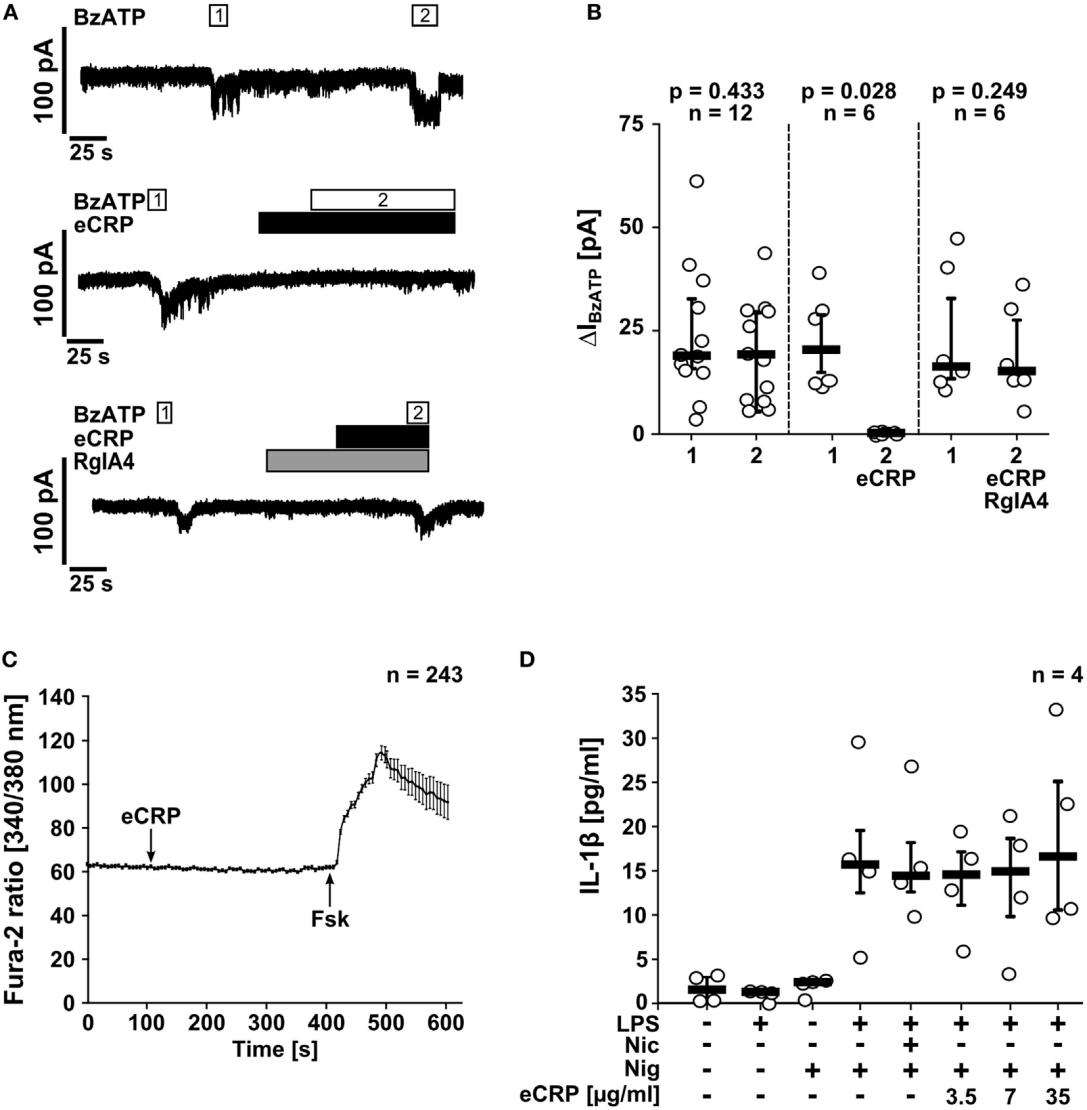
Purified human endogenous C-reactive protein (eCRP) suppresses BzATP-induced whole-cell currents *via* nAChRs. **(A)** BzATP-induced ion currents were detected by whole-cell patch-clamp measurements in lipopolysaccharide (LPS)-primed (1 µg/ml, 5 h) U937 cells. Repetitive current changes were provoked by two (1, 2) consecutive BzATP (100 µM) applications (upper panel). Application of eCRP (5 µg/ml) alone did not provoke ion currents but fully inhibited the response to BzATP (middle panel). The inhibitory effect of eCRP was antagonized by addition of the α9α10 nAChR-specific α-conotoxin RgIA4 (200 nM; lower panel). **(B)** Graphical presentation of the two consecutive BzATP-induced ion current changes (1, 2, ΔI_BzATP_). **(C)** [Ca^2+^]_i_ of LPS-primed U937 cells were recorded as Fura-2/AM (Fura-2) fluorescence intensity ratio of 340:380 nm excitation (mean ± SEM). Application of eCRP (5 µg/ml; indicated by arrow) did not cause significant alterations in [Ca^2+^]_i_ (values before eCRP compared to values obtained 300 s after eCRP application: *p* = 0.726). At the end of the experiments, a positive control for cell viability and the Ca^2+^ imaging setup was included: forskolin (Fsk, 40 µM, indicated by arrow) was applied to induce a cyclic adenosine monophosphate-triggered rise in [Ca^2+^]_i_. **(D)** The ATP-independent release of interleukin-1β (IL-1β) from LPS-primed monocytic U937 cells induced by nigericin (Nig; 50 µM) is neither inhibited by nicotine (Nic; 100 µM) nor by various concentrations of eCRP. Data are presented as individual data points, bar represents median, whiskers encompass the 25th to 75th percentile **(B,D)**. Wilcoxon signed-rank test **(B,C)** or Kruskal–Wallis followed by Mann–Whitney rank sum test **(D)**.

Next, we tested if eCRP induces ion fluxes in LPS-primed U937 cells. Application of eCRP (5 µg/ml) did not induce ion currents in whole-cell patch-clamp experiments (Figures 3A,B) and intracellular Ca^2+^ levels remained unchanged (Figure 3C). By contrast, BzATP (100 µM) induced a robust and repeatable current response in LPS-primed U937 cells (Figures 3A,B), as reported previously (30, 31). Remarkably, in the presence of eCRP, the BzATP-induced current responses were completely abrogated (Figures 3A,B). The inhibitory effect of eCRP was sensitive to the α-conotoxin RgIA4 (Figures 3A,B), confirming the involvement of nAChR subunits α9 and/or α10.

NLRP3 inflammasomes, in addition to extracellular ATP, assemble in response to other pro-inflammatory stimuli including pore-forming toxins (1). Here, the pore-forming bacterial toxin nigericin was used to investigate if eCRP also affects ATP-independent IL-1β release, which was clearly not the case (Figure 3D). Hence, stimulation of nAChRs with eCRP efficiently inhibits BzATP-induced ion currents in monocytic U937 cells but does not provoke canonical ion channel functions of nAChRs.

### eCRP Inhibits Inflammasome Activation in Human PBMC

**Figure 4:**
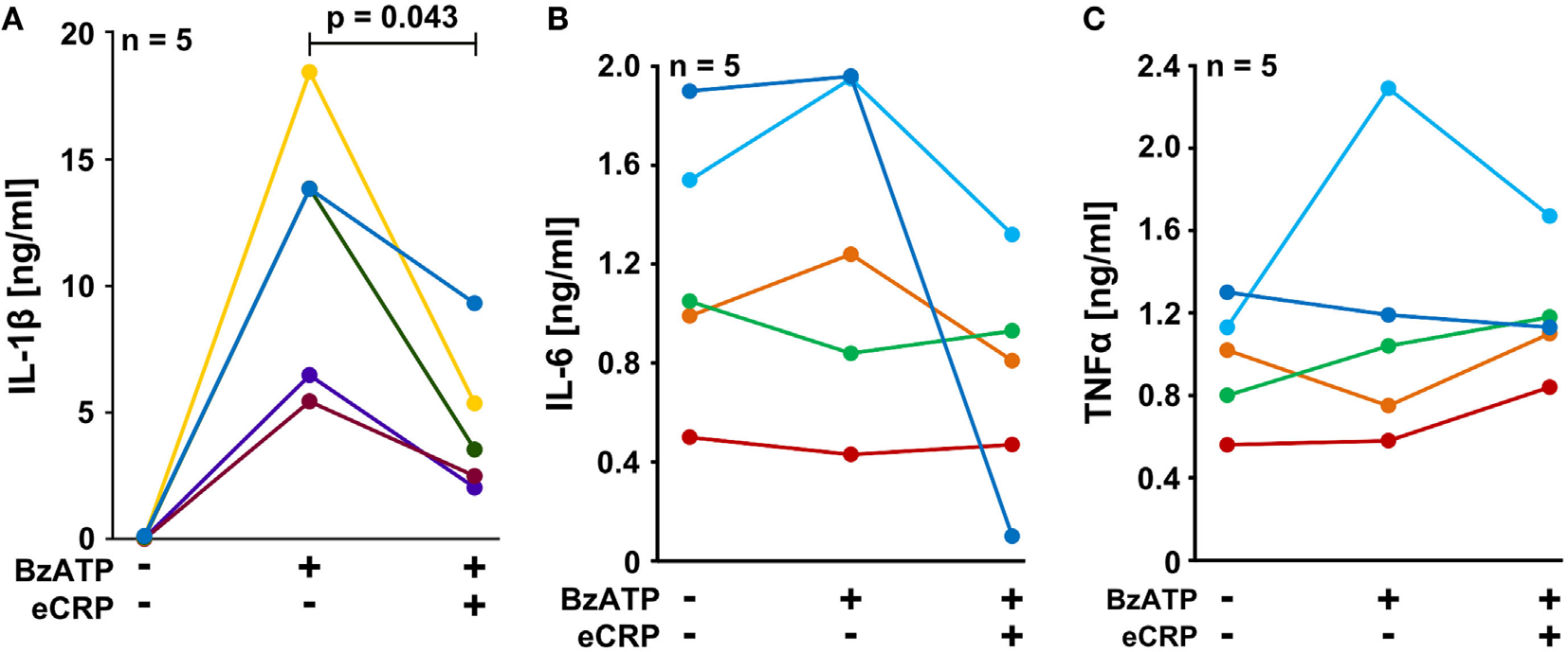
Purified human endogenous C-reactive protein (eCRP) inhibits BzATP-induced release of interleukin-1β (IL-1β) from human peripheral blood mononuclear cells (PBMC). PBMC freshly isolated from healthy volunteers were pulsed with lipopolysaccharide (5 ng/ml) during the process of PBMC isolation and cultured for 3 h. The cells were stimulated with 100 µM BzATP in the presence or absence of eCRP (5 µg/ml) for 30 min. **(A)** IL-1β, **(B)** IL-6, and **(C)** TNF-α were measured by ELISA in cell culture supernatants (*n* = 5). Data points obtained from individual blood donors are coded by different colors and connected by lines. Wilcoxon signed-rank test.

We performed experiments on the adherent fraction of freshly isolated PBMC from healthy human donors that were primed with a short pulse of LPS (5 ng/ml) during cell isolation. The spontaneous secretion of IL-1β by these cells was low, whereas a considerable amount of IL-1β was released in response to BzATP (100 µM). Indeed, eCRP (5 µg/ml) significantly attenuated the BzATP-induced release of IL-1β from these cells (Figure 4A), whereas the inflammasome-independent cytokines IL-6 and TNF-α (46) were neither induced by BzATP nor regulated by eCRP (Figures 4B,C). The concentrations of IL-6 and TNF-α released within 30 min after BzATP application are low and all apparent changes in response to BzATP or eCRP are not significant and probably random. We reported before, that almost no IL-18 is secreted by these cells in response to BzATP (30).

**Figure 5:**
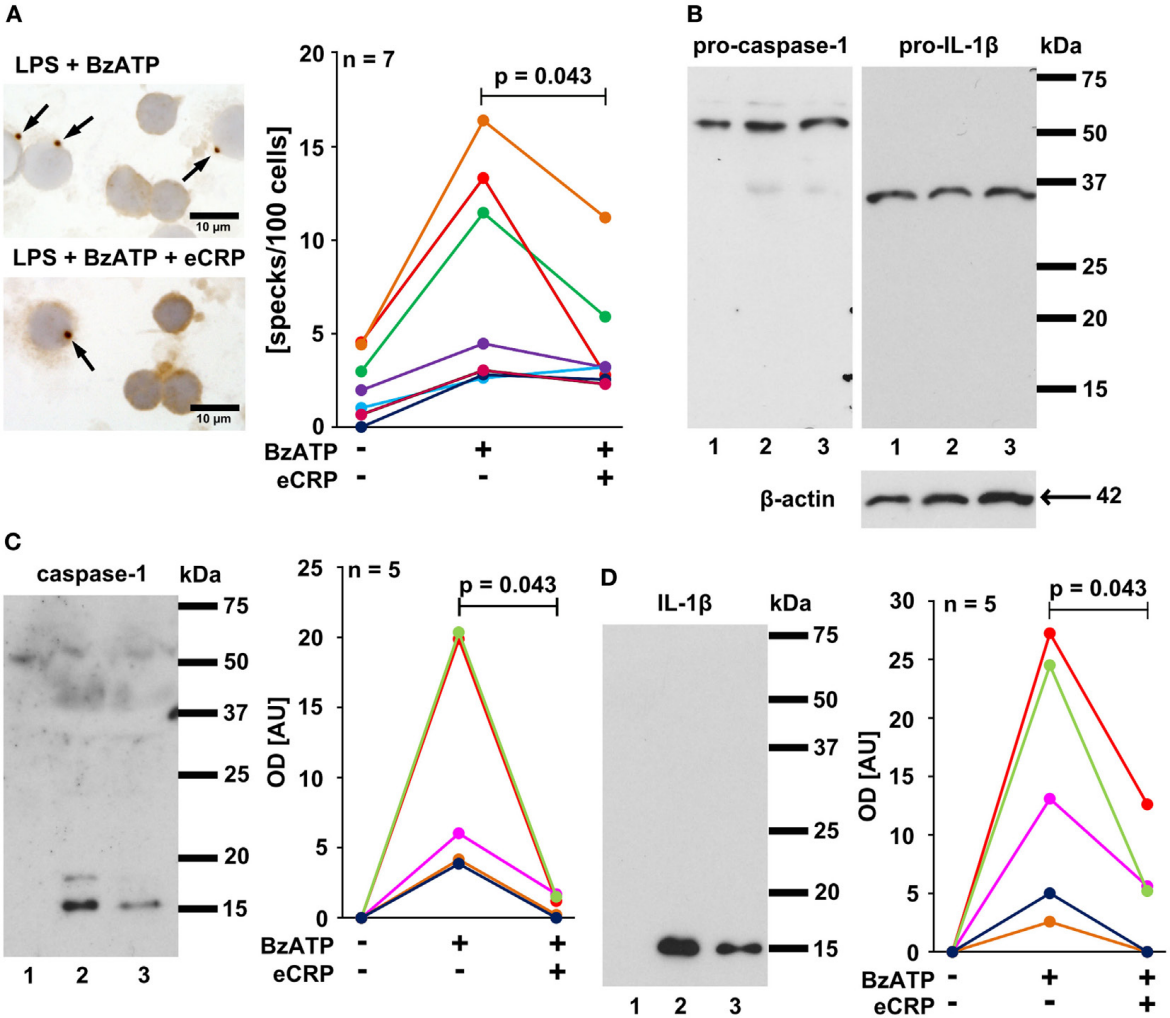
Purified human endogenous C-reactive protein (eCRP) inhibits BzATP-induced inflammasome activation, caspase-1 activation, and maturation of interleukin-1β (IL-1β) in human peripheral blood mononuclear cells (PBMC). **(A)** ASC specks (arrows) were detected by immunocytochemistry using antibodies directed to ASC (brown staining). Cell nuclei were lightly counterstained with hemalum. Specks were induced by treatment with BzATP and occurred less frequently when eCRP was added concomitantly (*n* = 7). **(B–D)** Western blot experiments were performed using antibodies that detect both, the pro-forms and the mature forms of caspase-1 and IL-1β, respectively (*n* = 5). Cell lysates and cell culture supernatants were investigated from lipopolysaccharide (LPS)-primed PBMC (1), LPS-primed PBMC stimulated with BzATP (2), and LPS-primed PBMC stimulated with BzATP in the presence of eCRP (3). **(B)** Pro-caspase-1 and pro-IL-1β were detected in cell lysates at about equal amounts irrespective of stimulation with BzATP, whereas no mature forms were present, although the antibodies used detect both the pro-forms and the native forms. Detection of β-actin was included as a loading control. **(C)** Only mature caspase-1 and **(D)** IL-1β were present in concentrated cell culture supernatants. Stimulation with BzATP induced the release of both proteins and the release was reduced by eCRP. Immunoreactivity was measured by densitometry [optical density (OD)]. Data points obtained from individual blood donors are coded by different colors and connected by lines. Wilcoxon signed-rank test.

Inflammasome activation can lead to the formation of large aggregates, so-called specks or pyroptosomes, that can be detected with antibodies directed to ASC (apoptosis-associated speck-like protein containing a caspase activation and recruitment domain) (1). To investigate if CRP inhibits inflammasome and caspase-1 activation in primary monocytic cells LPS-pulsed PBMC were challenged with BzATP (100 µM) in the presence and absence of eCRP (5 µg/ml). A significant increase in ASC speck formation was detected in LPS-primed PBMC in response to BzATP, which was largely suppressed by concomitant application of eCRP (Figure 5A). Pro-IL-1β and pro-caspase-1, but not their mature forms, were detected by Western blotting of cell lysates of LPS-primed PBMC stimulated with BzATP in the absence or presence of eCRP (Figure 5B). In concentrated supernatants, however, mature caspase-1 and IL-1β were detectable upon BzATP-treatment, and eCRP significantly reduced their amount (Figures 5C,D). We conclude that CRP suppresses inflammasome assembly, pyroptosome formation, activation of capase-1 and maturation of IL-1β.

### CRP and IL-1β Levels Negatively Correlate in Multiple Trauma Patients

**Figure 6:**
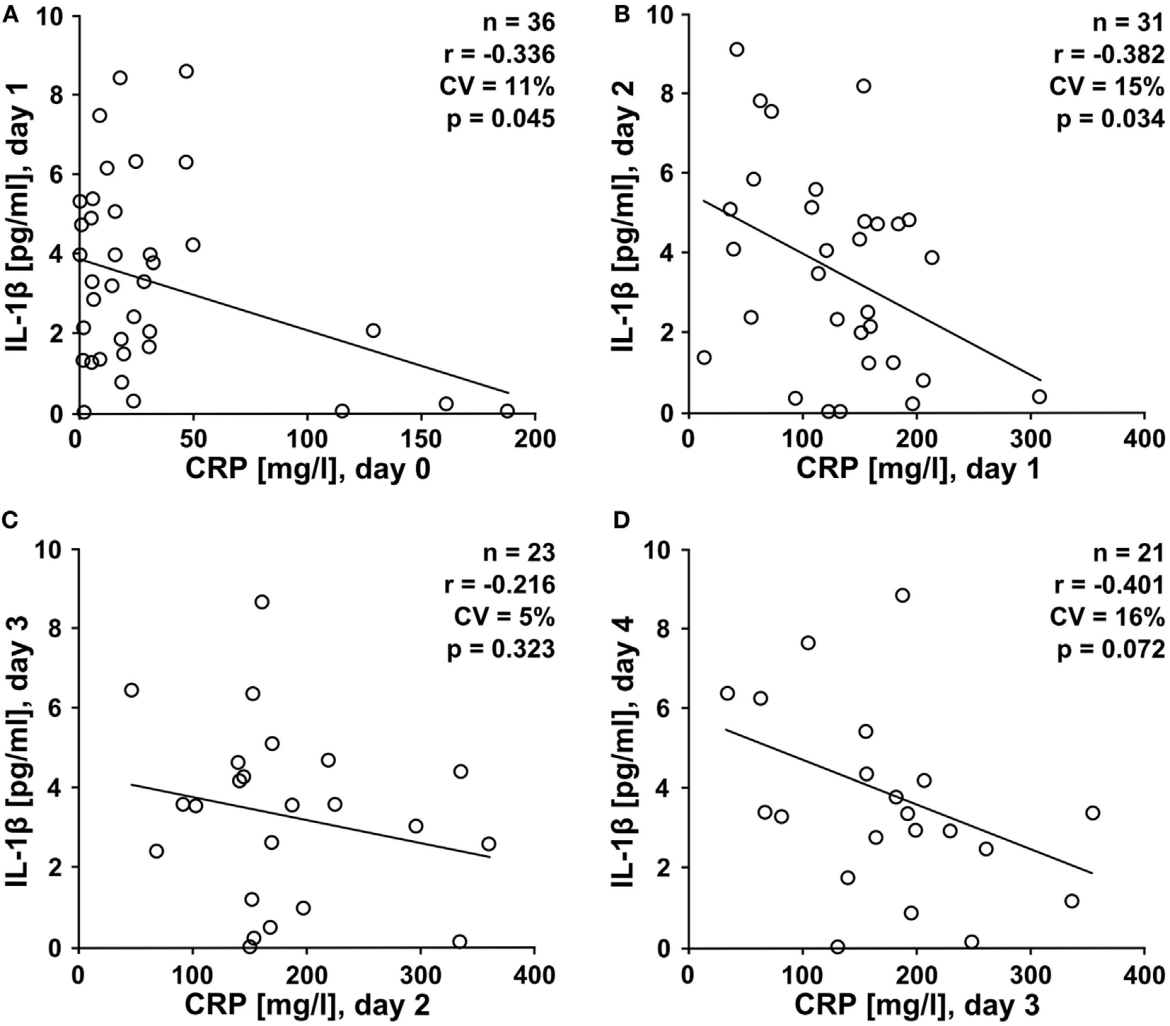
High levels of C-reactive protein (CRP) negatively correlate with low interleukin-1β (IL-1β) levels in trauma patients. A prospective clinical study was performed on patients suffering from multiple traumata. Plasma levels of IL-1β were measured at daily intervals from the day of admission (day 0) until day 4 after trauma and a correlation analysis was performed with CRP values obtained one day earlier. **(A)** IL-1β at day 1 versus CRP at day 0 (*n* = 36), **(B)** IL-1β at day 2 versus CRP at day 1 (*n* = 31), **(C)** IL-1β at day 3 versus CRP at day 2 (*n* = 23), and **(D)** IL-1β at day 4 versus CRP at day 3 (*n* = 21). Linear regression analysis, *r* = correlation coefficient, CV = coefficient of variation.

Our *in vitro* experiments led to the provocative hypothesis that elevated CRP levels protect against trauma-induced release of IL-1β into the circulation *in vivo*. We performed a prospective study on multiple trauma patients admitted to our hospital. Patient characteristics are summarized in Table 1. Plasma levels of IL-1β, IL-18, IL-6, TNF-α, and HMGB1 were measured in blood drawn at daily intervals until day 4 after admission. During the first 2 days, IL-1β levels negatively correlated with CRP values of the preceding day (Figures 6A–D), which is in line with our hypothesis. On day 4 after trauma, plasma levels of IL-6 (Figure S1 in Supplementary Material), IL-18 (Figure S2 in Supplementary Material), and TNF-α (Figure S3 in Supplementary Material) positively correlated with CRP values on day 3, whereas no correlation was seen for HMGB1 (Figure S4 in Supplementary Material). On days 0–2 after trauma, IL-1β levels did not correlate with disease severity acute physiology and chronic health evaluation score (APACHE II) and sequential organ failure assessment score (SOFA). In patients who remained on the ICU on days 3–4 after trauma a negative correlation of IL-1β levels with the SOFA score was seen (Table S1 in Supplementary Material). This subpopulation of patients typically suffers from a severe disease course. CRP levels did not correlate with APACHE II and SOFA score (Table S2 in Supplementary Material). Albeit statistically significant (*p* ≤ 0.05), the correlation coefficients (*r*) and, accordingly, the coefficients of variation (CV) (Figure 6; Figures S1–S4 and Tables S1 and S2 in Supplementary Material), do not prove causality.

## Discussion

**Figure 7:**
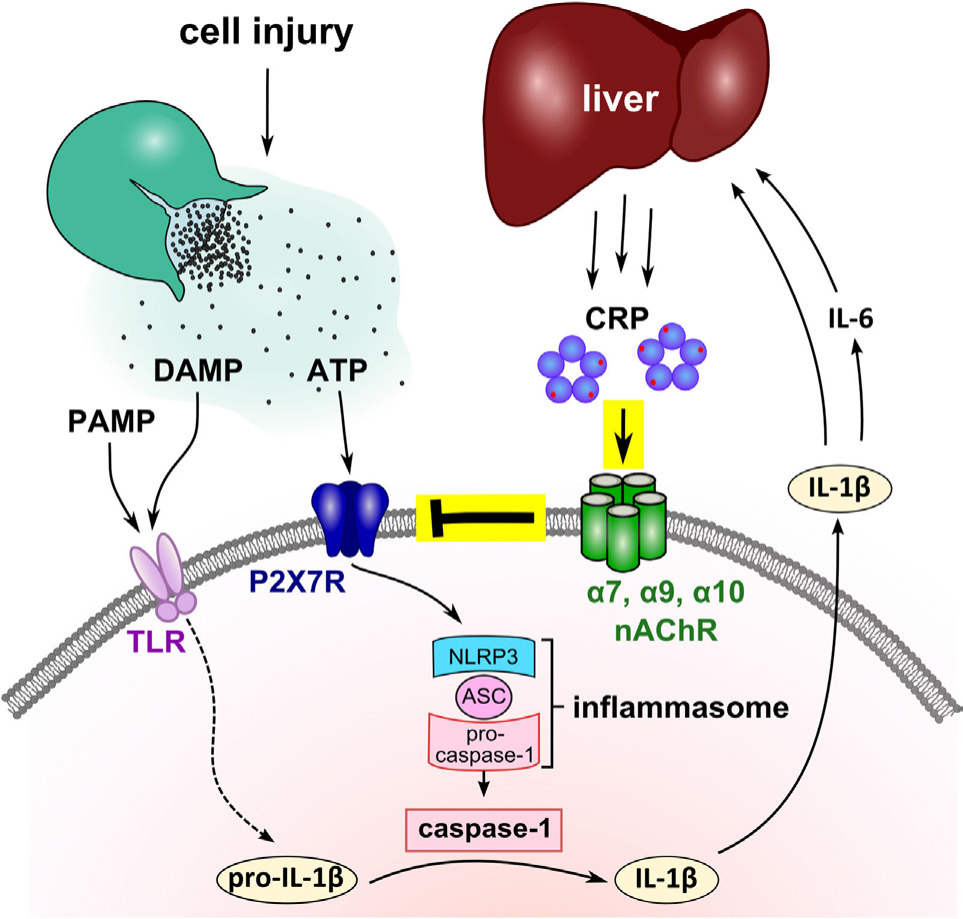
Suggested mechanism of the mutual control of C-reactive protein (CRP) and interleukin-1β (IL-1β) during trauma-associated sterile inflammation. Injury causes the release of cytoplasmic danger-associated molecular patterns (DAMP) and ATP, and frequently microbiota, a source of pathogen-associated molecular patterns (PAMP), get access to the damaged tissue. DAMP and PAMP can bind to pattern recognition receptors such as toll-like receptors (TLR) at monocytic cells and induce the synthesis of pro-IL-1β, the inactive cytoplasmic precursor of IL-1β. Extracellular ATP activates the P2X7R and leads to inflammasome assembly and caspase-1 activation. Activated caspase-1 cleaves pro-IL-1β and enables the release of mature bioactive IL-1β that in turn, induces IL-6. IL-1β and IL-6 activate the hepatic synthesis of CRP and blood levels of CRP quickly rise to 1,000-fold. We demonstrated that purified human endogenous CRP inhibits the response of P2X7R to ATP *via* nAChR subunits α7, α9, and α10. The function of CRP critically depends on its association with small molecules such as phosphocholine (red dots) that seem to mediate the interaction with nicotinic receptors. We suggest that the CRP-mediated control of ATP-induced IL-1β release is a negative feedback loop that controls excessive systemic inflammation in response to injury. Although the nAChR is illustrated as a pentamer, its molecular structure remains to be elucidated.

C-reactive protein is among the most frequently used clinical marker of inflammation, but its biological function is still a matter of debate. Here, we demonstrate that eCRP dose-dependently and efficiently inhibits the ATP-induced release of IL-1β from monocytic U937 cells at an IC_50_ of 4.9 µg/ml, corresponding to a marginally elevated blood CRP level (41). The activity of eCRP depends on the Ca^2+^-dependent interaction with a small molecule, presumably PC (14, 15), and on nAChR subunits α7, α9, and α10. Stimulation of monocytic nAChRs in turn fully inhibits ATP-induced ion currents (Figure 7). Furthermore, eCRP is also active in primary human adherent PBMC, where it inhibits inflammasome and caspase-1 activation. First clinical evidence from multiple trauma patients is in line with the results obtained *in vitro* but does not prove causality. As IL-1β and IL-6 are the main inducers of hepatic CRP synthesis during systemic inflammation and IL-1β is an important stimulus for IL-6 expression, we suggest that CRP is a negative feedback regulator of the ATP-dependent production of mature IL-1β by human monocytes (Figure 7).

CRP is commonly regarded as an opsonizing agent that binds to PC present on the surfaces of some pro- and eukaryotic pathogens as well as on dying cells (14, 15). Phagocytosis of these opsonized particles is mediated *via* complement fixation and binding of CRP to different Fc-receptors expressed by phagocytic cells (14, 15). The suggested role of CRP in host defense against infections is, however, in sharp contrast to a large body of literature showing that PC-containing cell surface molecules of bacteria as well as PC-modified proteins secreted by helminths exert anti-inflammatory functions leading to immune evasion and chronic colonization of the host (47, 48). We demonstrated before that PC-modified bovine serum albumin and PC-modified lipooligosaccharides from *Haemophilus influenzae* control the release of IL-1β *via* mechanisms resembling those of CRP (30). This suggests that pathogens with PC-modified surfaces hijacked the here described mechanism, which underscores the biological and medical relevance of CRP.

We identify nAChRs composed of subunits α7, α9, and α10 as receptors for eCRP. Our data corroborate the almost 30 years old finding, that CRP binds Ca^2+^-dependently to human monocytes with an EC_50_ of about 2.3 mg/l (49). Presumably, these authors measured the PC-dependent binding of CRP to monocytic nAChRs. We showed that PC-free CRP is inactive, and its activity can be reconstituted by adding low concentrations of PC that are in the range of those present in the serum of healthy persons (50). Thus, the interaction of eCRP with the ligand-binding site of nAChRs is probably mediated by CRP-bound PC. It has been shown before that endogenous human CRP can be laden with PC and other molecules with a PC group (14, 15, 29). In the same line, we demonstrated recently that high concentrations of free PC also inhibit the ATP-mediated release of IL-1β *via* nAChR (30–33), suggesting that CRP potentiates the effect of free PC. This is of importance *in vivo*, since typical human blood plasma concentrations of free PC are about 2 µM (50), while the IC_50_ of free PC is in the range of 10 µM, and 100 µM are needed for a full inhibition of the ATP-induced release of monocytic IL-1β (30).

Ligand-binding sites of conventional pentameric nAChRs that function as ligand-gated ion channels are formed by two neighboring nAChR subunits each and close upon binding of their cognate agonists (51). Interestingly, binding of a single ligand to only one of the five sites is sufficient for maximal ionotropic response of α7 nAChRs and additional binding sites enhance agonist sensitivity (52). The nAChR binding sites are dimensioned for classical nicotinic agonists but are certainly too small to enclose a macromolecule such as eCRP, suggesting that the structures of monocytic nAChRs are different. The activation of nAChR by a large molecule like eCRP is surprising but not unprecedented, as we demonstrated before that PC covalently bound to bovine serum albumin, PC-modified lipooligosaccharides and dipalmitoyl phosphatidylcholine function as potent agonists at unconventional monocytic nAChRs (30, 32). The IC_50_ values of PC-modified bovine serum albumin and lipooligosaccharides are also in the nanomolar range and hence, considerably lower than free PC, choline, or acetylcholine (30). If the structure of monocytic nAChRs differs from classical pentamers, it might be speculated that one eCRP pentamer interacts with several monocytic nAChRs. Once CRP binds to a nAChR, other receptors might be quickly recruited and activated. This hypothesis might explain the observed low IC_50_ values and the steep dose-response curve. This hypothesis may also apply to PC-modified albumin and to PC-lipooligosaccharides, because the PC-albumin investigated contained nine PC groups per BSA (30) and lipooligosaccharides tend to form micelles due to their amphiphilic structure. In contrast to the above-mentioned molecules with covalent PC modifications, it is also possible that eCRP delivers free PC to nAChRs because the affinities of PC to both molecules are in the same range (53). However, the considerably lower IC_50_ value of CRP compared to free PC (30) speaks against his theory. It may, however, explain, why eCRP acts as a silent agonist at canonical nAChRs heterologously expressed by *Xenopus* oocytes. Nevertheless, the structure of such unconventional nAChRs that exert metabotropic functions remains to be elucidated and it is even unclear if the leukocytic nAChR subunits form pentameric receptors at all.

Increasing evidence suggests that leukocytes in general respond to nicotinic stimuli *via* nAChRs with metabotropic responses, and no ligand-gated ion channel functions have been reported so far (44, 45, 54). A prominent example is the cholinergic regulation of the transcription and translation of pro-inflammatory cytokines by macrophages that is mediated *via* nAChR subunit α7 (54, 55). Here, we demonstrate that the P2X7R function of monocytic cells and the ATP-dependent release of IL-1β are fully inhibited by eCRP *via* stimulation of nAChRs containing subunits α7, α9, and α10. Nicotine, choline, and free PC exert similar effects albeit at much higher molar concentrations (30, 31, 33). The molecular signaling mechanism down-stream of monocytic nAChRs is currently under investigation.

Of note, eCRP neither induces ionotropic functions at monocytic nAChRs containing subunits α7, α9, and α10, nor at conventional nAChRs that were heterologously expressed by *Xenopus* oocytes. Similar results were shown before for free PC (31). Seemingly, eCRP is a novel agonist of nAChRs that exclusively induces metabotropic receptor functions and does not activate canonical ionotropic nAChR functions of excitable cells. Furthermore, we show that eCRP down-modulates the choline-induced ionotropic activity of heterologously expressed α9α10 nAChRs and provide evidence that CRP might be a silent agonist of nAChRs. As CRP and the nAChR subunits α7, α9, and α10 were highly conserved during evolution (14, 15, 51, 56), and mononuclear phagocytes are already present in primitive multicellular organisms (57), we speculate that the control of the P2X7R by nAChRs might be, in an evolutionary sense, older than neurotransmission.

We showed for the first time that in patients suffering from multiple traumata, IL-1β blood levels negatively correlated with preceding CRP levels. These results are in line with the hypothesis that the CRP-mediated control of IL-1β release is active *in vivo*, albeit they do not prove causality. According to Hill’s criteria for causation (58), “a small association does not mean that there is not a causal effect, though the larger the association, the more likely that it is causal.” Hill lists further criteria for causation including temporality, biological gradients, plausibility, and experimental evidence. Although, our data by and large meet with these criteria, more experimental and larger clinical multi-center studies are warranted, before we can dare to claim causality. The absence of a negative correlation of CRP levels with IL-18 and HMGB1 may be due to the ubiquitous expression of these inflammasome-dependent mediators, in contrast to IL-1β which is mainly produced by monocytes/macrophages (59).

Our data suggest that elevated CRP levels attenuate inflammatory diseases that are caused by ATP-induced inflammasome activation. It remains to be investigated if the here described mechanism also contributes to the protection against experimental inflammation in animals overexpressing human CRP (25–28). Interestingly, CRP was recently shown to impair dendritic cell development, maturation and function (60). This anti-inflammatory mechanism involves the inhibitory Fcγ receptor IIB (60). The potential clinical implications of these and our findings deserve further investigation, including a careful consideration of the known pro-inflammatory functions of CRP (14, 15, 22).

Several therapeutics targeting IL-1 or its receptor, among them the IL-1 receptor antagonist anakinra, the decoy receptor rilonacept and the neutralizing monoclonal anti-IL-1β antibody canakinumab, were tested in large trials but never reached the clinical arena for the treatment of SIRS (4). A major disadvantage of these approaches might be the general inhibition of IL-1β that should result in an impaired host defense against infections. By contrast, we showed that eCRP specifically inhibits the P2X7R-mediated response to extracellular ATP that is a danger signal mainly associated with mechanical cell damage (61). Viral, bacterial, and fungal pathogens activate numerous additional ATP-independent pathways of inflammasome activation and IL-1β maturation (1). Hence, we speculate that CRP predominantly inhibits trauma-associated release of IL-1β without preventing the IL-1β response to infection.

In conclusion, CRP efficiently inhibits ATP-dependent inflammasome activation and IL-1β release from human monocytic blood cells *in vitro*. This effect of CRP seems to depend on bound PC and activation of non-canonical nAChRs that efficiently inhibit the ion channel functions of monocytic ATP receptors. In the same line, we provide the first clinical evidence that elevated CRP levels might reduce systemic IL-1β release in patients suffering from multiple traumata.

## Ethics Statement

The local ethics committee at the University of Giessen approved all studies on primary human cells (approval No. 81/13). The study protocol for clinical sample collection (trial registration: DRKS00010991) was approved by the ethics committee of the medical faculty Giessen, Germany (No. 164/14) and performed in accordance with the Helsinki Declaration. All patients completed written informed consent prior to study entry.

## Author Contributions

KR, SS, ATZ, MK, JD, SH, SW, and AJH performed experiments and interpreted results; IA, MP, SR, AH, and CK recruited healthy donors and patients and interpreted results; IRK performed statistical analyses and interpreted results; WK, MS, K-DS, WP, JMM, and CK were involved in study design, interpretation of the results, and in writing; in addition, JMM provided seminal reagents; VG designed the study, interpreted data, and wrote the paper.

## Conflict of Interest Statement

Certain conotoxins, including RgIA4 have been patented by the University of Utah; JMM is an inventor on these patents. The other authors declare that the research was conducted in the absence of any commercial or financial relationships that could be construed as a potential conflict of interest.
