# Supplementary material for: C-Reactive Protein Stimulates Nicotinic Acetylcholine Receptors to Control ATP-Mediated Monocytic Inflammasome Activation
Source: Front Immunol. 2018 Jul 30;9:1604. doi: 10.3389/fimmu.2018.01604 (PMC6077200; doi:10.3389/fimmu.2018.01604)
Supplement: Supplementary file 1 [file Presentation_1.pdf]

C-reactive protein stimulates nicotinic acetylcholine receptors to control ATP-mediated monocytic inflammasome activation

Katrin Richter[1,*,†], Sabrina Sagawe[1,†], Andreas Hecker[1], Mira Küllmar[1], Ingolf Askevold[1], Jelena Damm[1], Sarah Heldmann[1], Michael Pöhlmann[2], Sophie Ruhrmann[2], Michael Sander[2], Klaus-Dieter Schlüter[3], Sigrid Wilker[1], Inke R. König[4,5], Wolfgang Kummer[6], Winfried Padberg[1], Arik J. Hone[7], J. Michael McIntosh[7-9], Anna Teresa Zakrzewicz[1], Christian Koch[2], and Veronika Grau[1]

[1]Laboratory of Experimental Surgery, Department of General and Thoracic Surgery, Justus-Liebig-University Giessen, German Centre for Lung Research (DZL), Giessen, Germany,
[2]Department of Anesthesiology and Intensive Care Medicine, Justus-Liebig-University Giessen, Giessen, Germany,
[3]Physiological Institute, Justus-Liebig-University Giessen, Giessen, Germany,
[4]Institute of Medical Biometry and Statistics, University of Luebeck, Luebeck, Germany,
[5]Airway Research Center North (ARCN), German Center for Lung Research (DZL), Giessen, Germany,
[6]Institute of Anatomy and Cell Biology, Justus-Liebig-University Giessen, German Centre for Lung Research, Giessen, Germany,
[7]Department of Biology, University of Utah, Salt Lake City, UT, United States,
[8]George E. Wahlen Veterans Affairs Medical Center, Salt Lake City, UT, United States,
[9]Department of Psychiatry, University of Utah, Salt Lake City, UT, United States,
[†]These authors have contributed equally to this work.

***Correspondence:**
Katrin Richter, Justus-Liebig-University of Giessen, Department of General and Thoracic Surgery, Laboratory of Experimental Surgery, Feulgenstr. 10 – 12, D-35392 Giessen; Tel. +49 641 985 45510; Fax: +49 641 985 44769;
E-mail address: Katrin.Richter@chiru.med.uni-giessen.de

Supplementary Material includes Figures S1 - S4, Table S1 and Table S2.

Figure S1

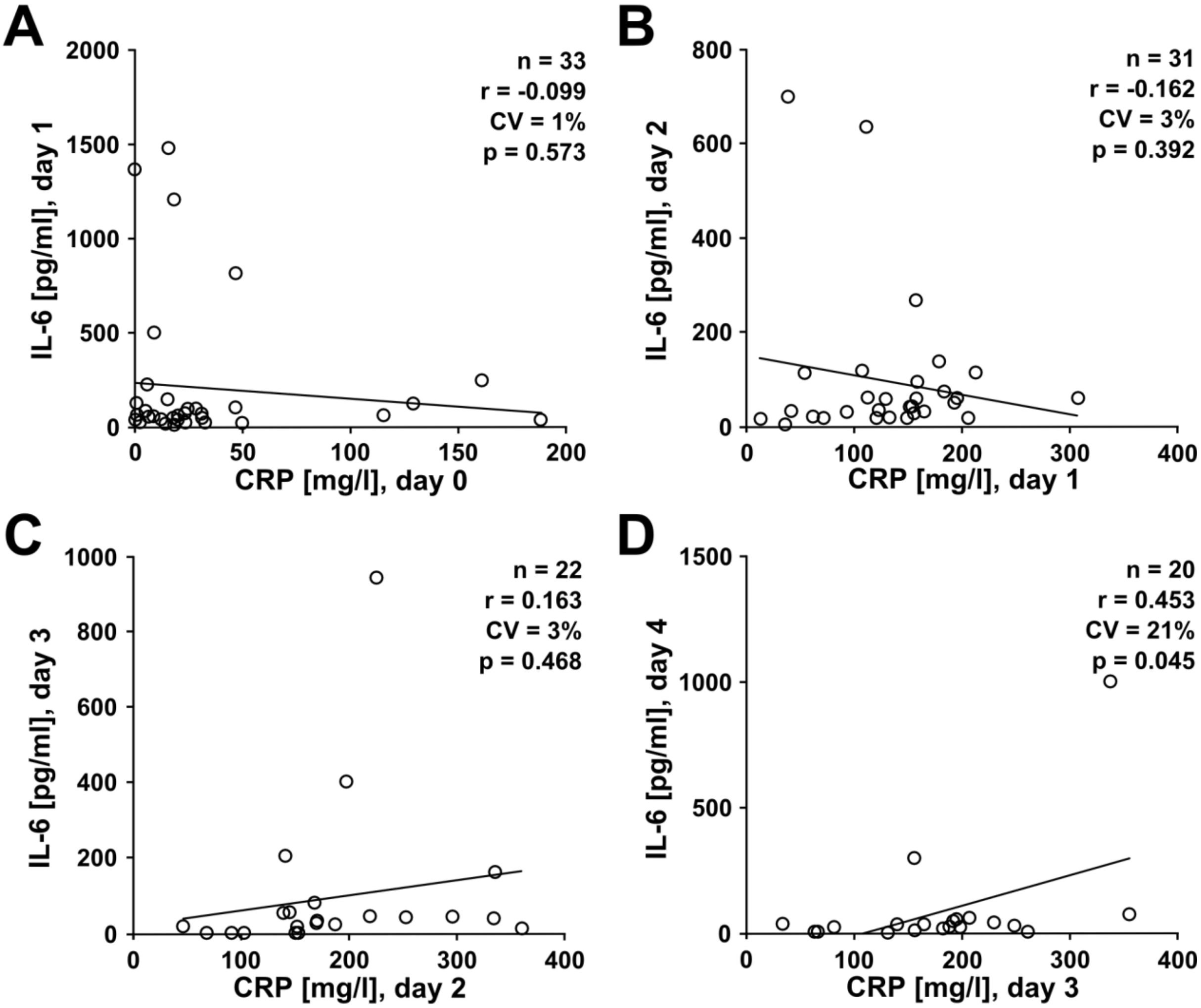

FIGURE S1. Levels of CRP do not negatively correlate with IL-6 levels in trauma patients. A monocentric prospective clinical study was performed on patients suffering from multiple traumata. Plasma levels of IL-6 were measured at daily intervals from the day of admission (day 0) until day 4 after trauma and a correlation analysis was performed with CRP values obtained one day earlier. **(A)** IL-6 at day 1 versus CRP at day 0, **(B)** IL-6 at day 2 versus CRP at day 1, **(C)** IL-6 at day 3 versus CRP at day 2, **(D)** a positive correlation was seen for IL-6 at day 4 versus CRP at day 3. Linear regression analysis, r = correlation coefficient, CV = coefficient of variation.

Figure S2

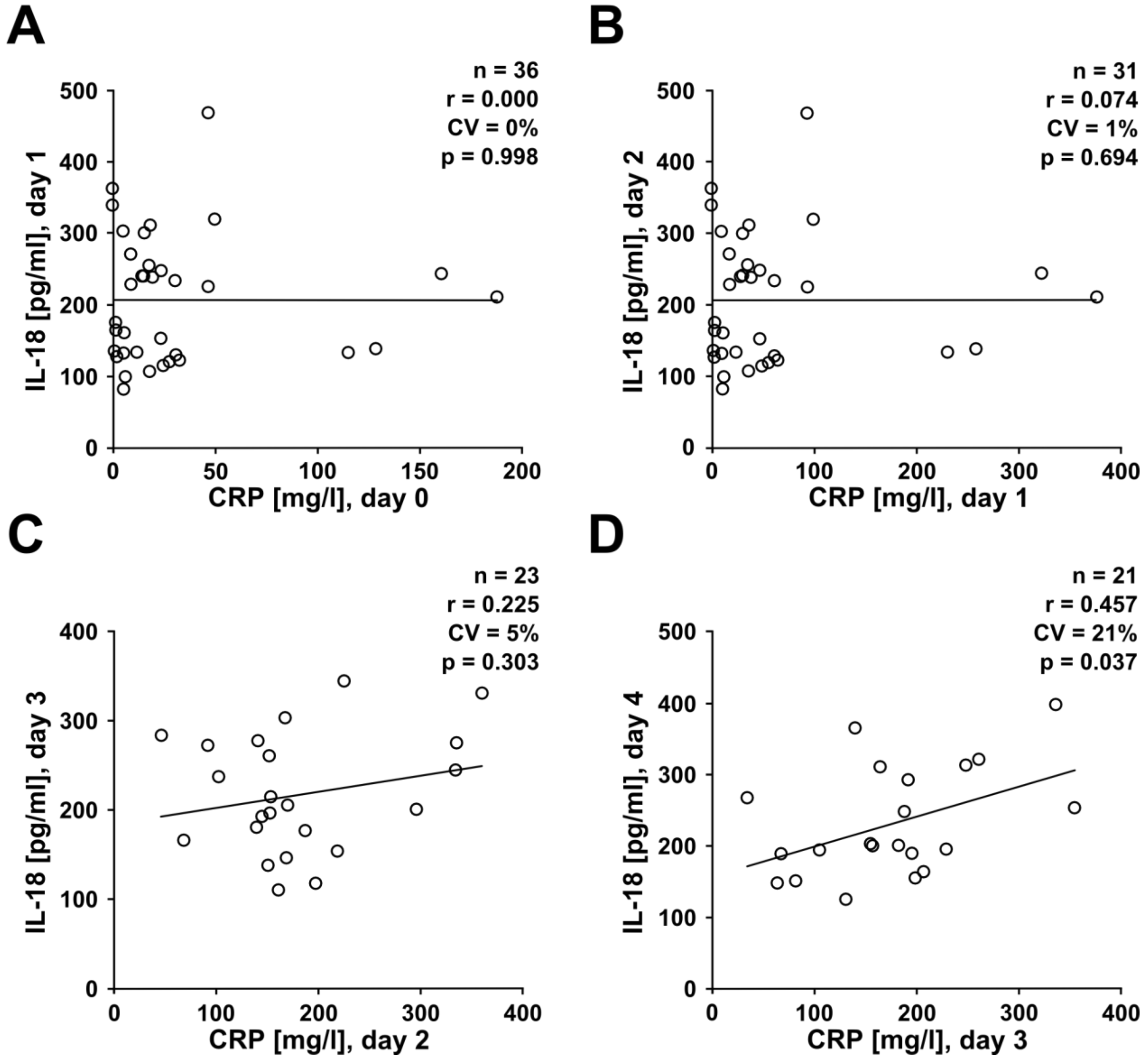

FIGURE S2. Levels of CRP do not negatively correlate with IL-18 levels in trauma patients. A monocentric prospective clinical study was performed on patients suffering from multiple traumata. Plasma levels of IL-18 were measured at daily intervals from the day of admission (day 0) until day 4 after trauma and a correlation analysis was performed with CRP values obtained one day earlier. **(A)** IL-18 at day 1 versus CRP at day 0, **(B)** IL-18 at day 2 versus CRP at day 1, **(C)** IL-18 at day 3 versus CRP at day 2, **(D)** a positive correlation was seen for IL-18 at day 4 versus CRP at day 3. Linear regression analysis, r = correlation coefficient, CV = coefficient of variation.

Figure S3

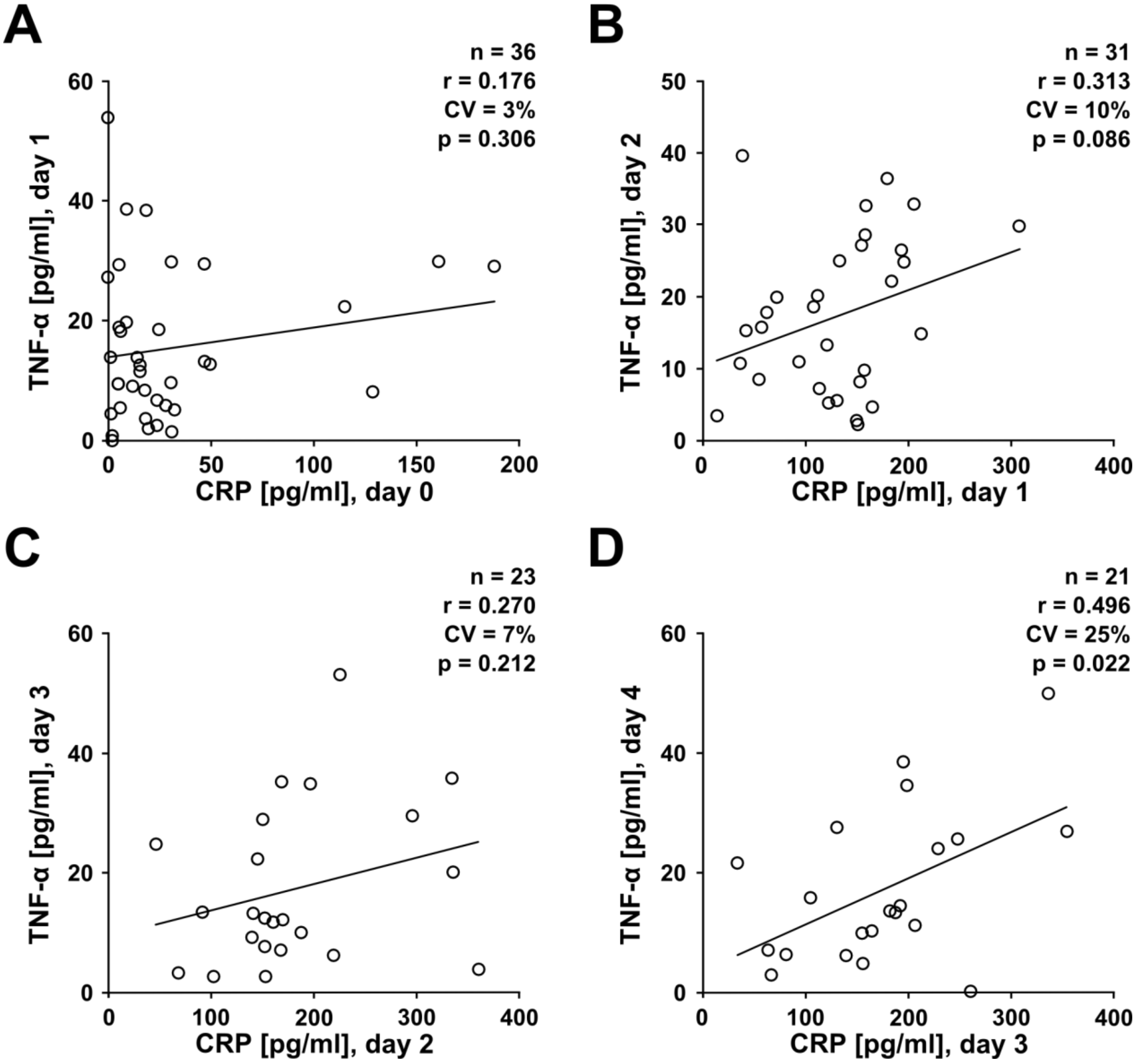

FIGURE S3. Levels of CRP do not negatively correlate with TNF-α levels in trauma patients. A monocentric prospective clinical study was performed on patients suffering from multiple traumata. Plasma levels of TNF-α were measured at daily intervals from the day of admission (day 0) until day 4 after trauma and a correlation analysis was performed with CRP values obtained one day earlier. **(A)** TNF-α at day 1 versus CRP at day 0, **(B)** TNF-α at day 2 versus CRP at day 1, **(C)** TNF-α at day 3 versus CRP at day 2, **(D)** a positive correlation was seen for TNF-α at day 4 versus CRP at day 3. Linear regression analysis, r = correlation coefficient, CV = coefficient of variation.

Figure S4

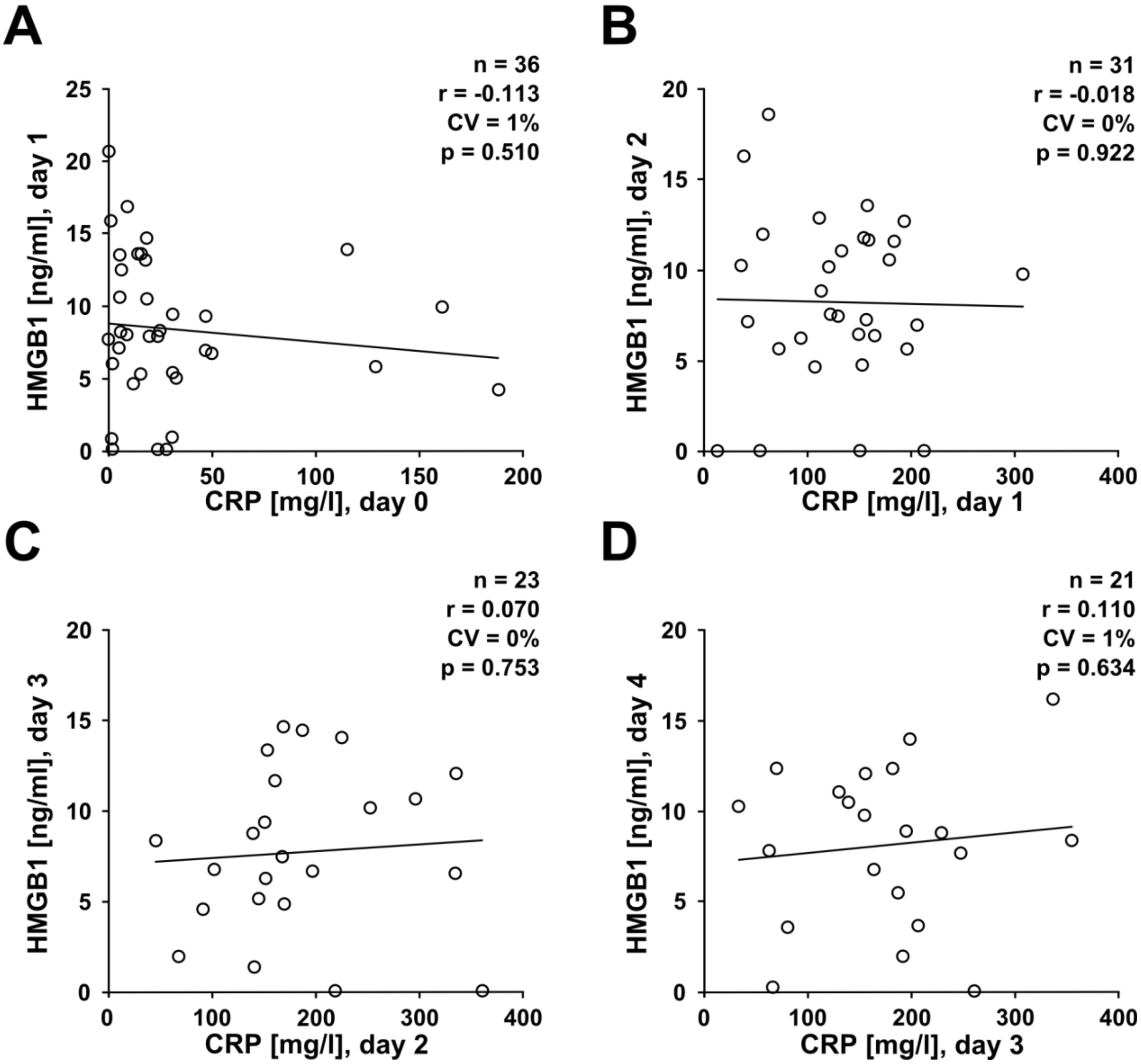

FIGURE S4. Levels of CRP do not negatively correlate with HMGB1 (high mobility group box 1 protein) levels in trauma patients. A monocentric prospective clinical study was performed on patients suffering from multiple traumata. Plasma levels of HMGB1 were measured at daily intervals from the day of admission (day 0) until day 4 after trauma and a correlation analysis was performed with CRP values obtained one day earlier. **(A)** HMGB1 at day 1 versus CRP at day 0, **(B)** HMGB1 at day 2 versus CRP at day 1, **(C)** HMGB1 at day 3 versus CRP at day 2, **(D)** HMGB1 at day 4 versus CRP at day 3. Linear regression analysis, r = correlation coefficient, CV = coefficient of variation.

TABLE S1. Correlation analyses of disease severity scores (day 0) with IL-1β levels (day 0 – day 4).

Variables					
**Score**	**IL-1β**	**n**	**r**	**CV**	**p**
APACHE II	day 0	38	-0.15	2%	0.38
SOFA	day 0	38	-0.23	5%	0.18
APACHE II	day 1	36	0.03	0%	0.86
SOFA	day 1	36	-0.11	1%	0.53
APACHE II	day 2	31	-0.13	2%	0.49
SOFA	day 2	31	-0.24	6%	0.21
APACHE II	day 3	23	-0.49	24%	0.022
SOFA	day 3	23	-0.53	28%	0.011
APACHE II	day 4	21	-0.56	31%	0.01
SOFA	day 4	21	-0.47	22%	0.035

APACHE II, acute physiology and chronic health evaluation score; n, number of patients; p, p-value; r, correlation coefficient; CV, coefficient of variation; SOFA, sequential organ failure assessment score.

TABLE S2. Correlation analyses of disease severity scores (day 0) with CRP levels (day 0 – day 4).

Variables					
**Score**	**CRP**	**n**	**r**	**CV**	**p**
APACHE II	day 0	38	0.035	0%	0.84
SOFA	day 0	38	0.12	1%	0.47
APACHE II	day 1	36	-0.029	0%	0.87
SOFA	day 1	36	0.091	1%	0.6
APACHE II	day 2	31	-0.023	0%	0.9
SOFA	day 2	31	0.026	0%	0.89
APACHE II	day 3	23	0.2	4%	0.38
SOFA	day 3	23	0.23	5%	0.29
APACHE II	day 4	21	0.063	0%	0.79
SOFA	day 4	21	0.08	1%	0.74

APACHE II, acute physiology and chronic health evaluation score; n, number of patients; p, p-value; r, correlation coefficient; CV, coefficient of variation; SOFA, sequential organ failure assessment score.
